# NK cell-mediated immunostimulatory effects of ethanol extract of *Morinda citrifolia* (noni) fruit

**DOI:** 10.1186/s12906-022-03700-3

**Published:** 2022-08-22

**Authors:** Haeyeop Kim, Laily Rahmawati, Yo Han Hong, Su-Young Choi, Jae Youl Cho

**Affiliations:** 1 Department of Integrative Biotechnology, Sungkyunkwan University, Suwon, 16419 Korea; 2 COSMAX NBT, INC, #504, Pangyo inno valley E, 255, Pangyo-ro, Seongnam, 13496 Korea; 3 Research Institute of Biomolecule Control and Biomedical Institute for Convergence at SKKU (BICS), Sungkyunkwan University, Suwon, 16419 Korea

**Keywords:** Noni, Immunostimulatory effects, NK cells, TNF-α

## Abstract

**Background:**

*Morinda citrifolia* (Noni) is a plant that has long been used in various products such as foods and cosmetics. Although noni has been known to have immunostimulatory activity, detailed mechanism at the cellular level has not been fully elucidated yet. In this study, we focused on understanding as to how noni fruit can positively stimulate body’s immune responses.

**Methods:**

To do this, an ethanol extract of noni fruit (Mc-fEE) was prepared and administered for 30 days to male C57BL/6 mice for in vivo experiment. NK cell activity and cytokine production level from Mc-fEE-treated mice were analyzed by flowcytometry, real-time PCR, and ELISA. Mc-fEE-triggered molecular events were detected from RAW264.7 cells and splenocytes using Western blotting and real-time PCR analyses.

**Results:**

The mRNA expression levels of cytokines such as interleukin families, interferon (IFN)-β, and tumor necrosis factor (TNF)-α were increased by Mc-fEE treatment in vitro and in vivo. Western blotting analysis showed that the phosphorylation levels of nuclear factor (NF)-κB and activator protein (AP)-1 subunits these were enhanced in Mc-fEE-treated RAW264.7 cells. In addition, according to in vivo experiments, it was considered that Mc-fEE can increase the population of splenic NK cells and subsequent upregulation of their cytotoxic activity against YAC-1 cells, a T- cell lymphoma.

**Conclusion:**

In this paper, we could confirm that Mc-fEE has remarkable immunostimulatory effects by activation and increase of the NK cell population.

**Supplementary Information:**

The online version contains supplementary material available at 10.1186/s12906-022-03700-3.

## Background

The immune system protects against various diseases [1, 2]. The immune system includes multiple proteins, cells, tissues, and organs and reacts to invading pathogens. Immune responses are either innate or adaptive [3, 4]. The innate immune system is the first line of defense against external stimuli, acting in a non-specific manner. Innate immunity refers to a system that recognizes specific nonself-pathogen-associated molecular patterns (PAMPs) through indicated pattern-recognition receptors (PRRs). This leads to the induction of downstream signaling pathways including activator protein (AP)-1 or nuclear factor-kappa B (NF-κB) pathways that ultimately regulate anti-pathogen responses [5, 6]. PAMPs are essential for the survival of microorganisms and are conserved highly for each species [7]. An example of well-known PAMPs includes lipopolysaccharide (LPS), the main constituent of the outer membrane of gram-negative bacteria, which interacts with PRR toll-like-receptor 4 (TLR4) [7].

The innate immune system includes epithelial barriers; phagocytes, including macrophages and dendritic cells; and complement cascades [8, 9]. Phagocytes mediate phagocytosis, plasma membrane-mediated engulfment of large particles; and professional phagocytes, mainly neutrophils and macrophages, remove pathogens or cell debris. Macrophages accumulate at the site of these cells through chemical signals (chemotaxis), and membrane attack complexes (MACs) are formed on the cell surface, resulting in cell lysis [10]. Then, agglutination, the conglomeration of infected cells for the easier antibody-mediated attack, occurs [11]. Macrophages may participate in an antitumor activity due to the production of effector molecules such as NO, TNF-α, and IL-1β [12, 13]. These macrophage-derived mediators have been recognized for their cytotoxic activities against cancer cells [14]. Natural killer (NK) cells are immune cells involved in innate immunity and possess various receptors that recognize abnormal cells, including infected cells or tumor cells [15–17]. The interaction between macrophages and NK cells is a critical initial line of defense against pathogens including viruses, bacteria, parasites, and fungi. In order to fight infections, NK cells must be recruited and activated to release cytokines and exert cytotoxicity at the sites of inflammation. Activation via soluble mediators such as IL-12 and IL-18, and stimulation by direct cell-to-cell contact, are the most common ways for macrophages to prime NK cells [14, 18, 19]. When antigens are not eliminated completely by the innate immune response, a more specific process, the adaptive immune response, is induced [20]. Adaptive immune responses also can be regulated by NK cells. Released cytokines such as TNF-α and IFN-γ from NK cells induce differentiation of CD4 + T cells to Th1 cells and maturation of APC cells. Mature dendritic cells induce the release of interleukins such as IL-12, which activates CD8 + T cells [21, 22].

*Morinda citrifolia* is a plant species in the genus *Morinda*, native to Southeast Asia including China, Australasia, and other South Pacific islands [23, 24]. This plant has been used in foods, cosmetics, and traditional medicines for its anti-fungal, anti-oxidative, or stamina-boosting effects [25, 26]. In addition, immunomodulatory activities of noni-derived components such as polysaccharide-rich substances from the fruit juice have been published by demonstrating their anti-cancer activities [27, 28], modulating the immune system via activating cannabinoid 2 receptors, and promoting the production of IFN-γ [29]. Other studies have demonstrated the effect of noni-derived components on the adaptive immune system, managed by B and T lymphocytes [30, 31]. Most of the immunomodulatory activities of this plant were studied under immunosuppression conditions [19, 32–34]. However, the detailed mechanism as to how this fruit can stimulate the immune system under normal healthy conditions is not yet fully addressed. Therefore, in this paper, we focused on exploring the immunoregulatory effect of an ethanol extract of the fruit of noni (*Morinda citrifolia*) (Mc-fEE) in the innate immune system under normal and healthy conditions by testing the activation levels of macrophage and NK cells. The molecular mechanisms of these effects were also investigated using in vitro and in vivo experiments.

## Methods

### Materials and antibodies

Mc-fEE **(**also called Nonitri®**)** and β-glucan were provided by COSMAXNBT Inc. (Korea). RAW264.7 cells (ATCC number TIB-71) were purchased from the American Type Culture Collection (ATCC) (Rockville, MD, USA) and YAC-1 (KCLB number 40160) cells were purchased from the Korean Cell Line Bank (Seoul, Korea). Roswell Park Memorial Institute (RPMI) 1640 media, antibiotics (penicillin–streptomycin solution), and phosphate buffered saline (PBS) were purchased from Hyclone (Logan, UT, USA). Stain buffer (FBS) and specific antibodies [FITC CD3, PE CD4, PE-Cy^TM^7 CD8, FITC CD11c, PE CD335 (NKp46), PE-Cy ^TM^7 F4/80] were purchased from BD Biosciences (Sparks, MD, USA). NK Cell Isolation Kit (mouse), buffer (0.5% BSA, 2 mM EDTA in PBS, pH 7.2), and autoMACS® Rinsing Solution (2 mM EDTA in PBS, pH 7.2) were purchased from Miltenyi Biotec (Bergisch Gladbach, Germany). Antibodies for p65, p50, c-Jun, c-Fos and their phosphor-specific antibodies were purchased from Cell Signaling Technology (Beverly, MA, USA). Antibody for β-actin was purchased from Santa Cruz Biotechnology Inc. (Santa Cruz, CA, USA). TNF-α ELISA kit (Quantikine™ ELISA, Cat No.: MTA00B) was purchased from R&D Systems (Minneapolis, MN, USA).

### Preparation of Mc-fEE and High-performance liquid chromatography (HPLC) analysis

Briefly, dried noni fruits were extracted with 10% ethanol at 80 °C for 4 h. Noni filtrates were evaporated, and the final yield of Mc-fEE was 20% (w/w). During the in vitro studies, the Mc-fEE stock solution was made by dissolving Mc-fEE stock with DMSO at a concentration of 100 mg/ml. When each experiment was performed, the stock solution was diluted to the desired final concentration of 0, 50 and 200  g/ml using the suitable culture medium. The Mc-fEE dissolved well in DMSO and was sterilized by heating at 85 °C for 20 min.

For standardization of this extract, high-performance liquid chromatography (HPLC) analysis was utilized to determine the phytochemical profile of Mc-fEE with a standard compound, deacetylasperulosidic acid as previously described [35].

### Cell culture and treatment

RAW264.7 cells (murine macrophage-like cells) and YAC-1 cells (a mouse T cell lymphoma) were cultured in RPMI 1640 media with 10% inactivated FBS, glutamine, and 1% antibiotics at 37 °C under 5% CO_2_. For in vitro experiments, Mc-fEE groups were pre-treated with Mc-fEE (50 mg/kg and 200 mg/kg), while the control (inducer alone) and normal (0  g/ml) groups were pre-treated with diluted DMSO in the culture medium.

### Animals

Male C57BL/6 mice (6–8 weeks old, 18–20 g) were purchased from OrientBio (Sungnam, Korea). Mice were caged in groups of 5 under a 12-h light and dark cycle and fed a pelleted diet and tap water ad libitum. For in vivo experiments, mice were orally administered with vehicle (0.5% CMC) or Mc-fEE (50 mg/kg and 200 mg/kg) once a day for 30 days. All animal studies were conducted according to the guidelines of the Institutional Animal Care and Use Committee of Sungkyunkwan University. (Suwon, Korea; Approval ID: SKKUIACUC2021-01–62-1).

### Cell viability test

The cell viability test was analyzed by an MTT assay as previously described [36]. RAW264.7 cells were seeded at a concentration of 1 × 10^6^ cells/ml per well in a 96-well-plate and incubated overnight. After incubation, media only as the negative control and the different concentrations of Mc-fEE, including diluted DMSO in the culture medium as 0  g/ml were treated, and cells were incubated for 24 h. Next, 100  l of supernatant of each well was removed, and 10  l of MTT solution (10 mg/ml in PBS, pH 7.4) was added for 3 h at 37 °C under 5% CO_2_. After incubation, 100  l of MTT stop solution (15% sodium dodecyl sulphate) was added, and the plate was incubated overnight. The absorbance was measured at 570 nm using a multi-reader Spectramax 250 (BioTex, Bad Friedrichshall, Germany), and the group values were normalized and compared to the negative control.

### Nitric oxide assay

RAW264.7 cells were seeded in a 96-well-plate at a concentration of 1 × 10^6^ cells/ml; after 24 h, cells were treated with or without polymyxin B (40  g/ml). After incubation for 30 min, Mc-fEE (0–200  g/ml) or LPS (1  g/ml) applied to each group. Cells were incubated for 24 h, and 100  l of supernatant was isolated to new 96-well-plates. Obtained supernatants were mixed with 100  l of Griess reagent, and the absorbance was measured at 540 nm using a multi-reader Spectramax 250 (BioTex, Bad Friedrichshall, Germany).

### Enzyme-linked immunosorbent assay (ELISA)

RAW264.7 cells were plated in a 96-well-plate at a concentration of 1 × 10^6^ cells/ml; after 24 h, cells were treated with the different concentrations of Mc-fEE (50 and 200  g/ml), including diluted DMSO in the culture medium as 0  g/ml for an additional 24 h. Next, 100  l of supernatant was used to measure the amount of TNF-α using an ELISA kit (R&D Systems, Minneapolis, MN, USA) according to the manufacturer’s instructions.

### mRNA analysis using quantitative real-time Polymerase Chain Reaction (PCR)

RAW264.7 cells were treated with the different concentrations of Mc-fEE (50 and 200  g/ml), including diluted DMSO in the culture medium as 0  g/ml, and harvested after 24 h. Mice were orally administered with vehicle (0.5% CMC) or Mc-fEE (50 mg/kg and 200 mg/kg), and spleens were isolated from the mice. Total RNA from cell lysates and splenocytes was acquired using TRIzol reagent according to the manufacturer’s instructions. cDNA was synthesized from total RNA (1  g) as previously reported [37]. The mRNA expression levels of IFN-β, TNF-α, IFN-γ, and interleukins (6, 10, 1β, 12b) were obtained using quantitative real-time PCR with SYBR Premix Ex Taq (Takara, Japan) on a thermal cycler (Bio-Rad, USA). The gene expression results were normalized as the ratio of optimal density relative to GAPDH. The primers used in this experiment are listed in Table 1.

**Table 1:** Primer sequences used in a quantitative real-time PCR

Gene	Direction	Sequences (5’ to 3’)
IL-6	Forward Reverse	GAC AAA GCC AGA GTC CTT CAG AGA CTA GGT TTG CCG AGT AGA TCT C
IFN-β	Forward Reverse	AAG AGT TAC ACT GCC TTT GCC ATC CAC TGT CTG CTG GTG GAG TTC ATC
TNF-α	Forward Reverse	TGC CTA TGT CTC AGC CTC TT GAG GCC ATT TGG GAA CTT CT
IL-1β	Forward Reverse	GTG AAA TGC CAC CTT TTG ACA GTG CCT GCC TGA AGC TCT TGT TG
IL-12b	Forward Reverse	TGG AGC ACT CCC CAT TCC TA GAG CTT GCA CGC AGA CAT TC
IFN-γ	Forward Reverse	TGG CTG TTT CTG GCT GTT ACT GTT GCT GAT GGC CTG ATT GTC
GAPDH	Forward Reverse	CAC TCA CGG CAA ATT CAA CGG CAC GAC TCC ACG ACA TAC TCA GCA C

### Western blotting analysis

RAW264.7 cells (2.5 × 10^6^ cells/ml) were seeded in 3-cm plates and incubated overnight at 37 °C under 5% CO_2_. After incubation, cells were treated with either Mc-fEE (200  g/ml), β-glucan (50  g/ml), or diluted DMSO in the culture medium as a control for the indicated time (0–60 min). Cells were isolated with cold PBS and prepared for western blotting analysis as previously described [38]. Proteins were analyzed using specific antibodies diluted in 3% BSA buffer (1:2500) and detected using an ECL reagent.

### Flow cytometry analysis

Male C57BL/6 mice (15 mice per group) were orally administrated with Mc-fEE (0—200 mg/kg) once a day for 30 days by using an oral zonde needle. Spleens were isolated and ground with RPMI 1640 media. Ground splenocytes were suspended in stain buffer (1% BSA, 0.1% sodium azide). The cell suspension was stained with the specific antibodies for 40 min at 4 °C, and the florescence was detected using a CytoFLEX Flow Cytometer (Beckman Coulter Life Sciences, Indianapolis, IN, USA).

### NK cell isolation and LDH cytotoxicity assay

Male C57BL/6 mice (15 mice per group) were orally administrated with Mc-fEE (0—200 mg/kg) once a day for 30 days. Total spleens were obtained and ground with RPMI 1640 media. Ground splenocytes, 3 × 10^7^ cells, were aliquoted into e-tubes and centrifuged at 300 g for 10 min. The supernatants were removed, and the cells were suspended in 120  l of buffer (0.5% BSA, 2 mM EDTA in PBS, pH 7.2). Then, 30  l of NK Cell Biotin-Antibody Cocktail was added to each e-tube, and cells were pipetted to be resuspended. After incubation using a rotator at 4 °C for 5 min, 1 ml of buffer was added to each e-tube, the tube was centrifuged at 300 g for 10 min, and the supernatants were removed. After adding 240  l of buffer, 60  l of Anti-Biotin MicroBeads was added and incubated for 10 min at 4 °C using a rotator. LS columns were inserted into the MACS Separator and washed with 2 ml of autoMACS® rinsing solution (2 mM EDTA in PBS, pH 7.2). Buffer was added so that the volume of the cell suspension was at least 500  l, and NK cells were obtained through column flow. After centrifuging at 3000 rpm for 5 min, the obtained NK cells were suspended in RPMI 1640 media and plated at 10^5^ cells/100  l and 5 × 10^4^ cells/100  l in round bottom 96-well-plates. YAC-1 cells were co-cultured with NK cells at a concentration of 10^4^ cells/100  l. The cells were incubated at 37 °C under 5% CO_2_ for 6 h. The round bottom 96-well-plates were centrifuged at 250 g at room temperature for 10 min, and the supernatants were transferred to a new 96-well-plate. Next, 100  l of reaction solution (catalyst: dye solution = 1: 45) was added to each well and incubated at room temperature for 5–15 min while blocking light. After that, 50  l of 1 N HCl was added to stop the reaction, and absorbance was measured at 490 nm.

$$\mathrm{Cytotoxicity} (\%)=\frac{\mathbf{A}-\mathbf{l}\mathbf{o}\mathbf{w} \mathbf{c}\mathbf{o}\mathbf{n}\mathbf{t}\mathbf{r}\mathbf{o}\mathbf{l}}{\mathbf{h}\mathbf{i}\mathbf{g}\mathbf{h}\mathbf{o}, \mathbf{c}\mathbf{o}\mathbf{n}\mathbf{t}\mathbf{r}\mathbf{o}\mathbf{l}-\mathbf{l}\mathbf{o}\mathbf{w} \mathbf{c}\mathbf{o}\mathbf{n}\mathbf{t}\mathbf{r}\mathbf{o}\mathbf{l}}\times 100$$

A: [effector – target cell mix] – [effector cell control]

high control: target cells + Triton X-100

low control: only target cells

### Statistical analysis

For statistical comparisons, a Student’s *t*-test was used to determine the statistical significances of the difference between values for the multiple experimental and control groups. Data were expressed as the mean ± standard error, and the results were obtained from at least three independent experiments of 5 samples (enzyme assay), 5 samples (in vitro experiments), and 15 mice per group (in vivo experiments). A *p*-value < 0.05 was considered statistically significant.

## Results

### Mc-fEE shows immunostimulatory effects in vitro

**Fig. 1:**
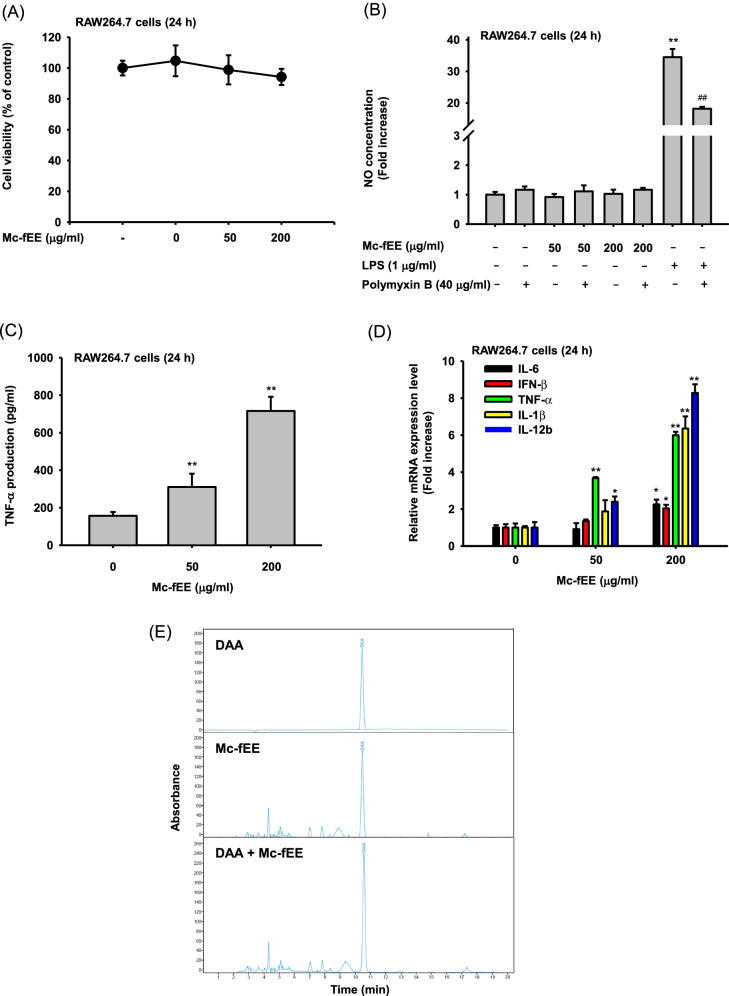
Immunostimulatory effects of Mc-fEE in vitro.** a** Mc-fEE (0–200  g/ml) was used to treat RAW264.7 cells for 24 h, and cell viability was measured by MTT assay. **b** Polymyxin B (40  g/ml) was used to treat RAW264.7 cells for 30 min, Mc-fEE (0–200  g/ml) or LPS (1  g/ml) was used to treat these cells for 24 h, and NO production was measured by NO assay. **c** TNF-α production level was detected by ELISA in supernatants of Mc-fEE (0–200  g/ml)-treated RAW264.7 cells. **d** RAW264.7 cells treated with Mc-fEE (0–200  g/ml) were used to detect various immune response-related pro-inflammatory cytokines, including IL-6, IFN-β, TNF-α, IL-1β, and IL-12b. **e** The phytochemical profiles were detected by using HPLC analysis with comparing the standard component, deacetylasperulosidic acid. Results (b-d) are expressed as mean ± SD (*n* = 4–5) and similar experimental data were obtained from an additional independent experiment performed under same conditions. *: *p* < 0.05, **: *p* < 0.01 compared to the normal group. ##: *p* < 0.01 compared to LPS alone

To check the immunostimulatory effects in vitro, we first conducted multiple experiments with RAW264.7 cells. First, to test cytotoxicity of this extract, we treated RAW264.7 cells with Mc-fEE for 24 h at concentrations of 50 g/ml and 200  g/ml using a MTT assay (Fig. 1a) [39]. The result shows that there was no interference of cell viability up to 5% upon treatment with Mc-fEE in RAW264.7 cells. Next, it was found that nitric oxide (NO) level was not increased by Mc-fEE at concentrations of 50 and 200  g/ml, while LPS, an endotoxin as toll-like receptor 4 (TRL4) ligand, significantly upregulated the NO level. In addition, to confirm whether Mc-fEE can show stimulatory activity in vitro by contaminated endotoxin, we also co-treated Mc-fEE with polymyxin B, an antibiotic for gram-negative bacteria and is widely used to determine endotoxin contamination [40]. Upon treatment with Mc-fEE at concentrations of 50 and 200 g/ml and polymyxin B, it was revealed that polymyxin B did not significantly affect NO production of Mc-fEE, while it significantly inhibited the production of NO by treatment of positive LPS control (Fig. 1b). The results of this NO assay suggest the absence of endotoxin. Additionally, we performed an ELISA to determine the protein expression level of TNF-α, and the results showed that TNF-α production level was increased significantly by Mc-fEE treatment at concentrations of 50 g/ml and 200 g/ml (Fig. 1c). Also, the mRNA expression levels of various cytokines were detected by quantitative real-time PCR. Mc-fEE at concentrations of 200 g/ml significantly upregulated the expression levels of several cytokines, including IL-6, IFN-β, TNF-α, and IL-1β, and IL-12b, which could induce NK cell activation (Fig. 1d). This finding seems to confirm a possibility that Mc-fEE can display immunostimulatory effects. Finally, we obtained the phytochemical profiles of Mc-fEE by using high-performance liquid chromatography (HPLC) analysis. As shown in Fig. 1e, by comparing with the standard content, we determined that Mc-fEE contains deacetylasperulosidic acid (DAA), an iridoid compound mainly found in plants [41]. This compound is well known to have various bioactivities, including anti-inflammatory, anti-cancer, and antioxidant effects [42, 43]. Therefore, these results imply that Mc-fEE possesses immunostimulatory effects, through the increase of cytokines with immunostimulatory property.

### Mc-fEE mediates the NF-κB and AP-1 pathway to have immunostimulatory effects

**Fig. 2:**
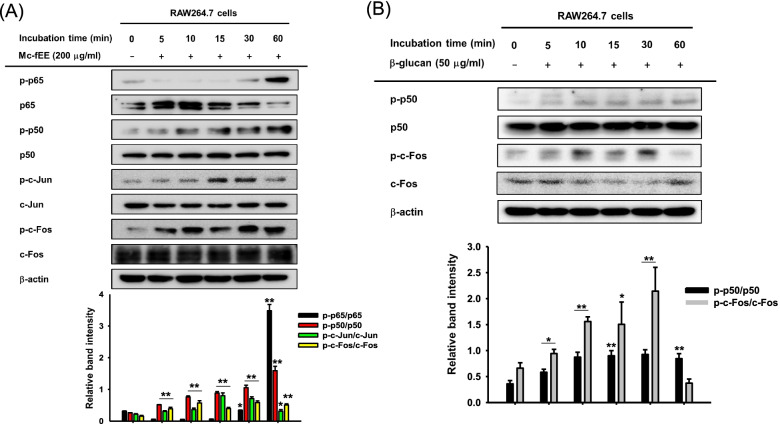
NF-κB and AP-1 pathway-mediated immunostimulatory effects of Mc-fEE. **a** Mc-fEE was used at a concentration of 200  g/ml to treat RAW264.7 cells for indicated times. Western blotting analysis was conducted with total and phosphorylated antibodies of NF-κB subunits (p65, p50) and AP-1 subunits (c-Jun, c-Fos). **b** Total and phosphorylated p50 and c-Fos were determined in β-glucan-treated RAW264.7 cells in a time-dependent manner. The data are expressed as means + SD of three independent experiments. *: *p* < 0.05, **: *p* < 0.01 compared to the normal group

To determine how Mc-fEE can increase the levels of pro-inflammatory cytokines, we determined specific transcription factor levels by immunoblotting analysis of this extract by comparison with the activity of β-glucan, a family member of β-D-glucose polysaccharides [44]. This molecule is present in the cell wall of fungi or bacteria and is known to induce an immune response by interacting with receptors of macrophages [45]. The levels of phosphorylation of p65 and p50 (NF-κB subunits) as well as c-Jun and c-Fos (AP-1 subunits) were increased in Mc-fEE-treated cells (Fig. 2a). Moreover, as shown in Fig. 2b, the levels of phosphorylation of p50 and c-Fos were also enhanced by β-glucan (50 g/ml) as a positive control. These data strongly indicate that the immunostimulatory efficacy of Mc-fEE in vitro could be a result of enhanced activity of NF-κB and AP-1 pathways, involved in the regulation of immune response.

### Mc-fEE increases the expression levels of NK cells population in the spleen

**Fig. 3:**
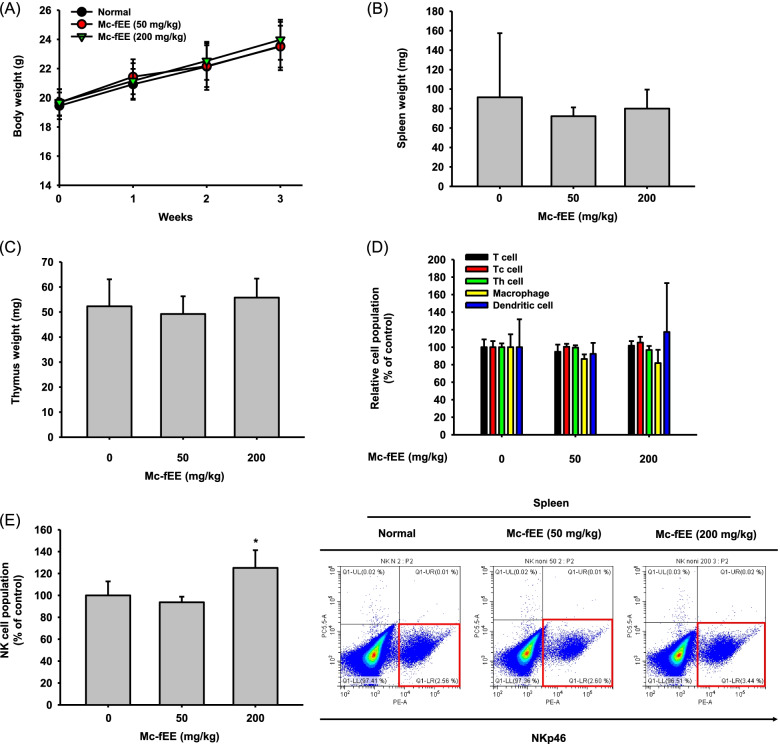
In vivo effects of Mc-fEE on populations of NK cells in mice spleens. **a** Body weights of Mc-fEE (0–200 mg/kg)-treated mice were measured once a week for 4 weeks. **b, c** Spleen or thymus weights were obtained from Mc-fEE (0–200 mg/kg)-treated mice. **d** T cells CD3 + , Tc cells CD3 + /CD4-/CD8 + , Th cells CD3 + /CD4 + /CD8-, macrophages F4/80 + , and dendritic cells CD11c + were isolated from the spleens of orally treated Mc-fEE (0–200 mg/kg) mice. The populations of cells were detected by flow cytometry analysis. **e** Populations of NK cells NKp46 + from orally treated Mc-fEE mice spleens were measured by flow cytometry analysis and plotted. The data are expressed as mean ± SD (*n* = 15). *: *p* < 0.05 compared to the normal group

We next tested the immune-stimulating efficacy of Mc-fEE in vivo. For 4 weeks, mice were administered orally with 50 mg/kg or 200 mg/kg doses of Mc-fEE, and at both dosages, the body weights of the mice were increased similarly to that of the untreated group (Fig. 3a). This suggests that Mc-fEE is not toxic in vivo. In agreement, there were no significant differences in spleen and thymus weights under Mc-fEE administration conditions (Fig. 3b, 3c). The in vivo immunostimulatory effects of Mc-fEE were evaluated by measuring the populations of immune cells, especially NK cells, as depicted in Fig. 3. We performed flow cytometric analysis and confirmed the population of several immune cells including Tc (cytotoxic T cells), Th (T helper cells), NK, and dendritic cells, as well as macrophages in the spleen of Mc-fEE-treated mice (shown in Supplementary Fig. 1 and Fig. 3d). Among these various types of immune cells, Mc-fEE significantly increased the population of NK cells (Fig. 3e). These results seem to suggest that Mc-fEE has the immune-enhancing ability by increasing the population of NK cells, which contribute to a vital role in early defense against infection.

### Mc-fEE shows immunostimulatory effects ex vivo

**Fig. 4:**
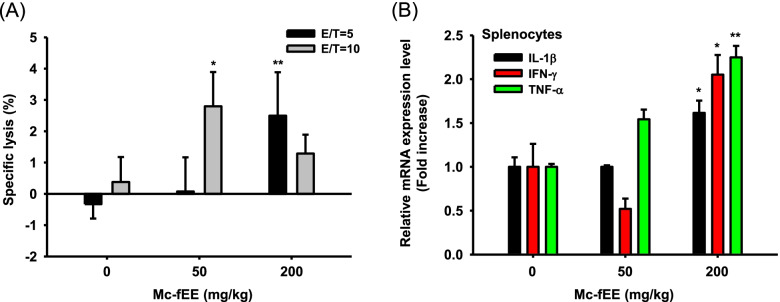
Immunostimulatory effects of Mc-fEE in vivo by promoting NK cell activities. **a** Specific lysis levels of YAC-1 cells by activated NK cells from Mc-fEE-treated mice spleens were measured by cell-mediated LDH assay. **b** Relative mRNA expression levels of IL-1β, IFN-γ, and TNF-α were detected from Mc-fEE-treated mice splenocytes using quantitative real-time PCR. The data are expressed as mean ± SD (*n* = 3) with an additional experiment under same conditions. *: *p* < 0.05, **: *p* < 0.01 compared to the normal group

Last, we conducted an additional experiment to determine whether NK cell activity can be also enhanced by Mc-fEE. For testing this purpose, we established a condition in which Yac-1 cell lysis level was evaluated after coculturing Yac-1 cells, a mouse T cell lymphoma, and NK cells prepared from spleens of Mc-fEE-treated mice [46], as depicted in Fig. 4a. Experiments were carried out a ratio, NK cells to YAC-1 cells of 5:1 or 10:1. NK cells from Mc-fEE-treated mice had greater cytotoxicity to YAC-1 cells compared to untreated mice (Fig. 4a). Indeed, Mc-fEE (200 mg/kg) showed more effective for cytotoxicity activity against YAC-1 cells in lower ratio of NK cells to YAC-1 cells (E/T = 5), whereas lower dose of Mc-fEE (50 mg/kg) had significant cytotoxic activity in E/T = 10. Furthermore, splenocytes from Mc-fEE-treated mice were obtained and the increased levels of immunostimulatory factors were identified at the mRNA level. Supporting the in vitro result (Fig. 1d), it was validated that the expression levels of IL-1β, IFN-γ, and TNF-αwere significantly upregulated at doses of 200 mg/kg (Fig. 4b). Among them, in particular, IL-1β is known to be a factor that plays a role in activating NK cells [47]. Through these factors, it was confirmed that the cytotoxic ability of NK cells can be increased by Mc-fEE.

## Discussion

We investigated the immunostimulatory role of Mc-fEE in vitro using RAW264.7 cells and in vivo using mice. Since Mc-fEE is relatively common natural product-derived extract which can be easily contaminated with environmental LPS, we first evaluated the effect of Mc-fEE on the production of NO during the exposure of polymyxin B (40 μg/ml), an inhibitor of gram‐negative bacteria [48]. This approach can help us to exclude the further possibility that the immunostimulatory effect of Mc-fEE can be caused by contaminated endotoxin (LPS). The results of NO assay strongly suggested that there was no contamination of endotoxin. Next, it was observed that Mc-fEE itself can improve the immunological function of murine macrophage RAW264.7 cells, according to the measurement of expression of various cytokines from the macrophage-like cells. Thus, Mc-fEE dose-dependently upregulated the mRNA expression levels of IL-6, IFN-β, TNF-α, IL-1β, and IL-12b (Fig. 1D). Of them, interestingly, the induction effect of these cytokines by Mc-fEE at 200  g/ml was maximized in IL-12 expression (Fig. 1D). IL-12, consisting of a heterodimeric structure of IL-12a and b, stimulates the functions of T cells. IL-12 is known to increase the expression of TNF-α and IFN-γ, and the cytotoxic activity of NK cells [49], implying that enhancement of TNF-α production and NK cell activity might be related to the strong induction of IL-12b by Mc-fEE.

We also confirmed that the mRNA expression levels of IL-1β and TNF-α were remarkably upregulated when murine macrophage RAW264.7 cells were exposed to Mc-fEE in vitro. Not only in mRNA level, but also the extracellular production level of TNF-α protein was also found to be increased (Fig. 1). In general, IL-1β and TNF-α are well-known cytokines acting as representative mediators of tumor cytostasis [50, 51]. Macrophages are the major cells secreting these cytokines in their anti-tumor immunity [14, 52]. Considering with these and other works [14, 32, 35, 53, 54], our data apparently indicate that Mc‐fEE becomes an immunostimulatory remedy targeting to macrophages, since the cells can boost L-1β and TNF-α production.

The mechanism for this increase in cytokine expression was also examined, and one finding was that the activation levels of NF-κB and AP-1 subunits, as assessed by their phosphorylation level [55], was increased during the treatment of Mc-fEE, as shown in the case of β-glucan (Fig. 2A and B). Namely, it was observed that phosphorylation of p50 and p65, NF-κB subunits, and as c-Jun and c-Fos, AP-1 subunits, was strongly upregulated by Mc-fEE (Fig. 2). This suggests that upstream signaling events for the activation of NF-κB and AP-1 pathways [56] might be triggered by Mc-fEE exposure. Thereafter, it is speculated that the survival, activation, and differentiation of multiple innate immune cells (eg., macrophages and NK cells) and T cells might be controlled by transcriptional gene products managed by AP-1 and NF-κB [57, 58]. In addition, the activation of AP-1 pathway was also known to induce the differentiation of T regulatory cells that suppress autoimmune diseases by suppressing self-reactivity [59].

Regarding in vivo study, the NK cell population, measured by flow cytometric analysis, in the spleens of mice treated with orally administered Mc-fEE was also revealed to be enhanced (Fig. 3), in accordance with our previous studies using *M. citrifolia* fruit water extract [35]. NK cells and cytotoxic T lymphocytes (CTLs) are generally involved with its anti-cancer properties [33, 60]. For evaluating whether Mc-fEE can enhance killing activity of NK cells, the level of LDH released from lysed YAC-1 cells was determined, as previously reported [61]. Several papers have described that immunostimulatory activity of noni contributes to its anticancer activity [19, 33, 62]. Subsequently, we also hoped to test whether Mc-fEE can upregulate cytotoxic activity of NK cells against YAC-1 cells. In fact, the increased level of LDH in the Mc-fEE-treated group compared to the untreated group was obviously observed (Fig. 4), implying that the cytotoxic capability of NK cells can be further acquired by Mc-fEE. 50 mg/kg of Mc-fEE had a greater effect on cytotoxicity in E/T = 10, whereas 200 mg/kg of Mc-fEE showed more effectiveness in E/T = 5. In particular, a higher ratio of NK cells to YAC-1 cells of Mc-fEE (200 mg/kg) showed a decreased activity of its cytotoxicity. Conceivably, it is indicating that at higher dose of Mc-fEE (200 mg/kg), a lower ratio of NK cells seemed to be required to target YAC-1 cells. Thus, this finding might suggest that the activation of NK cells might be depending on the levels of some immunostimulatory components. Moreover, it is considerable that more activated conditions of NK cells by higher dose of Mc-fEE seem to need less numbers of target cells (Yac-1) for their cytotoxicity. Furthermore, in agreement with findings in macrophage-like cells, Mc-fEE also enhanced the expression levels of IL-1β, TNF-α, and IFN-γ in splenocytes. Taken together, the in vivo and ex vivo results strongly suggest that Mc-fEE has immunostimulatory effects under normal immune response conditions by elevating the activity of NK cells and increasing the expression levels of cytokines in the spleens of mice. Our results also seem to provide supportive evidence as to how the fruit of noni can display anti-tumor and the immunomodulatory activities [19, 32–34].

## Conclusion

**Fig. 5:**
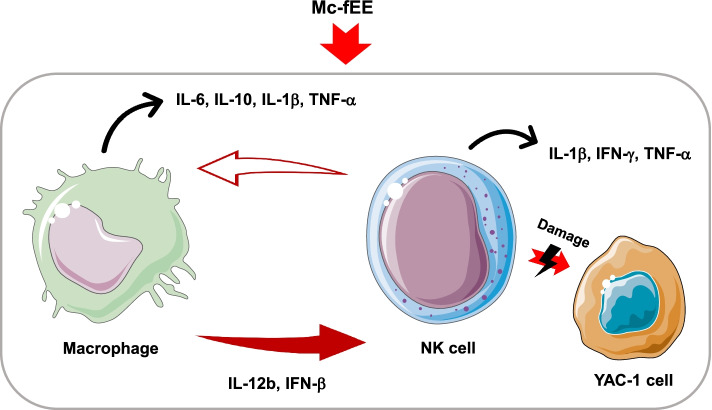
Scheme for Mc-fEE-mediated immunostimulatory effects

We found that Mc-fEE had an immunostimulatory effect by enhancing the population and activation of NK cells in vitro and in vivo. It was revealed that Mc-fEE (0–200  g/ml) can induce the mRNA expression levels of cytokines such as IL-6, IFN-β, TNF-α, IL-1β, and IL-12b in RAW264.7 cells via the activation of NF-κB and AP-1 pathways. Moreover, Mc-fEE (0–200 mg/kg) was observed to increase the population of NK cells and upregulate their cytolytic activity, which was demonstrated when isolated NK cells from Mc-fEE-treated mice were co-cultured with YAC-1 cells, and confirmed through LDH assay. Therefore, these results strongly suggest that Mc-fEE has immunostimulatory effects by elevating the activity of NK cells as summarized in Fig. 5.

## Supplementary Information

**Additional file 1:** 

## Data Availability

All data generated or analyzed during this study are included in this published article.
