# Supplementary material for: NK cell-mediated immunostimulatory effects of ethanol extract of Morinda citrifolia (noni) fruit
Source: BMC Complement Med Ther. 2022 Aug 22;22:222. doi: 10.1186/s12906-022-03700-3 (PMC9394078; doi:10.1186/s12906-022-03700-3)
Supplement: Supplementary file 1 — Additional file 1: [file 12906_2022_3700_MOESM1_ESM.pptx]

## Slide 1
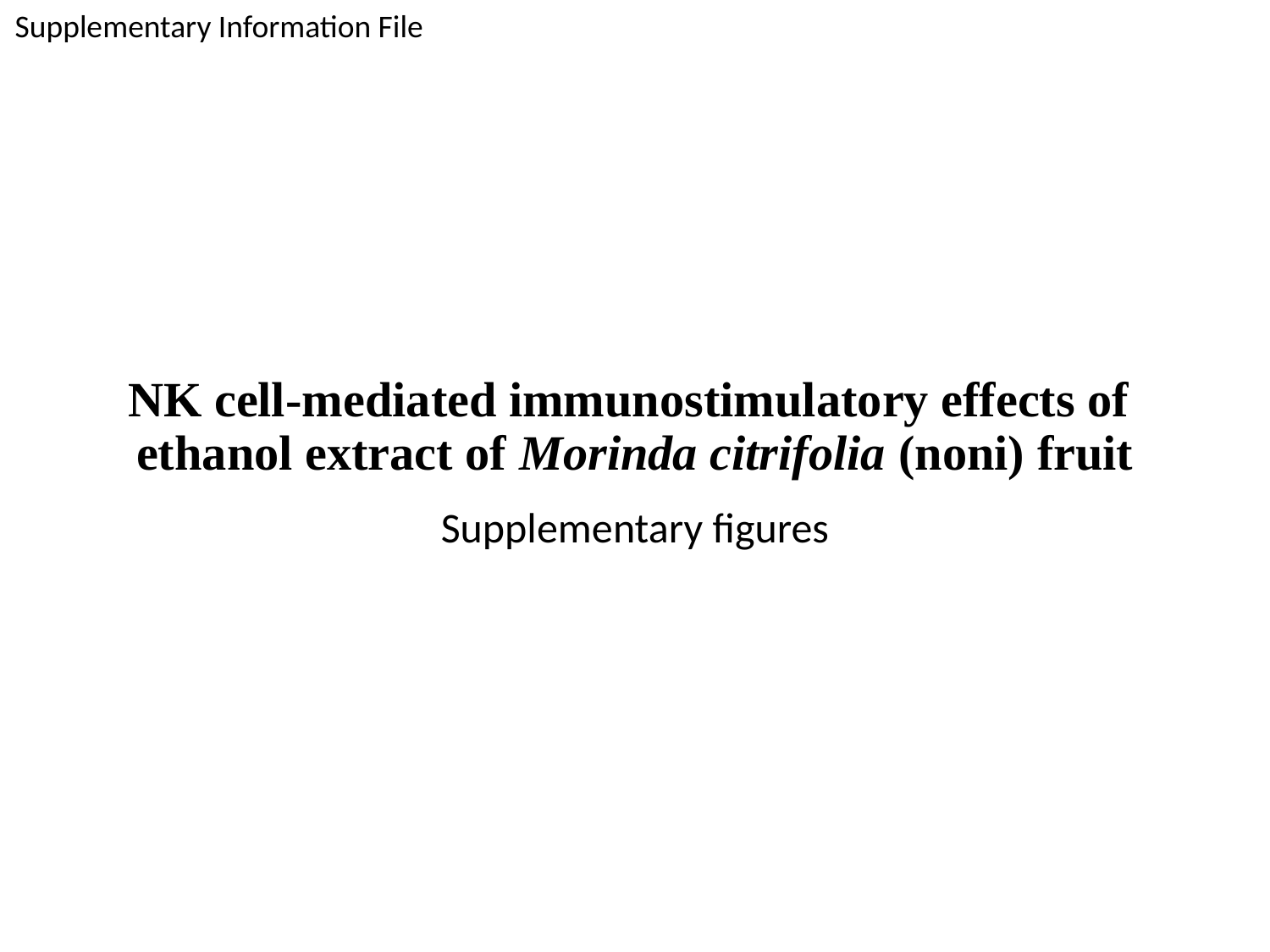

Supplementary Information File
# NK cell-mediated immunostimulatory effects of ethanol extract of Morinda citrifolia (noni) fruit
Supplementary figures

## Slide 2
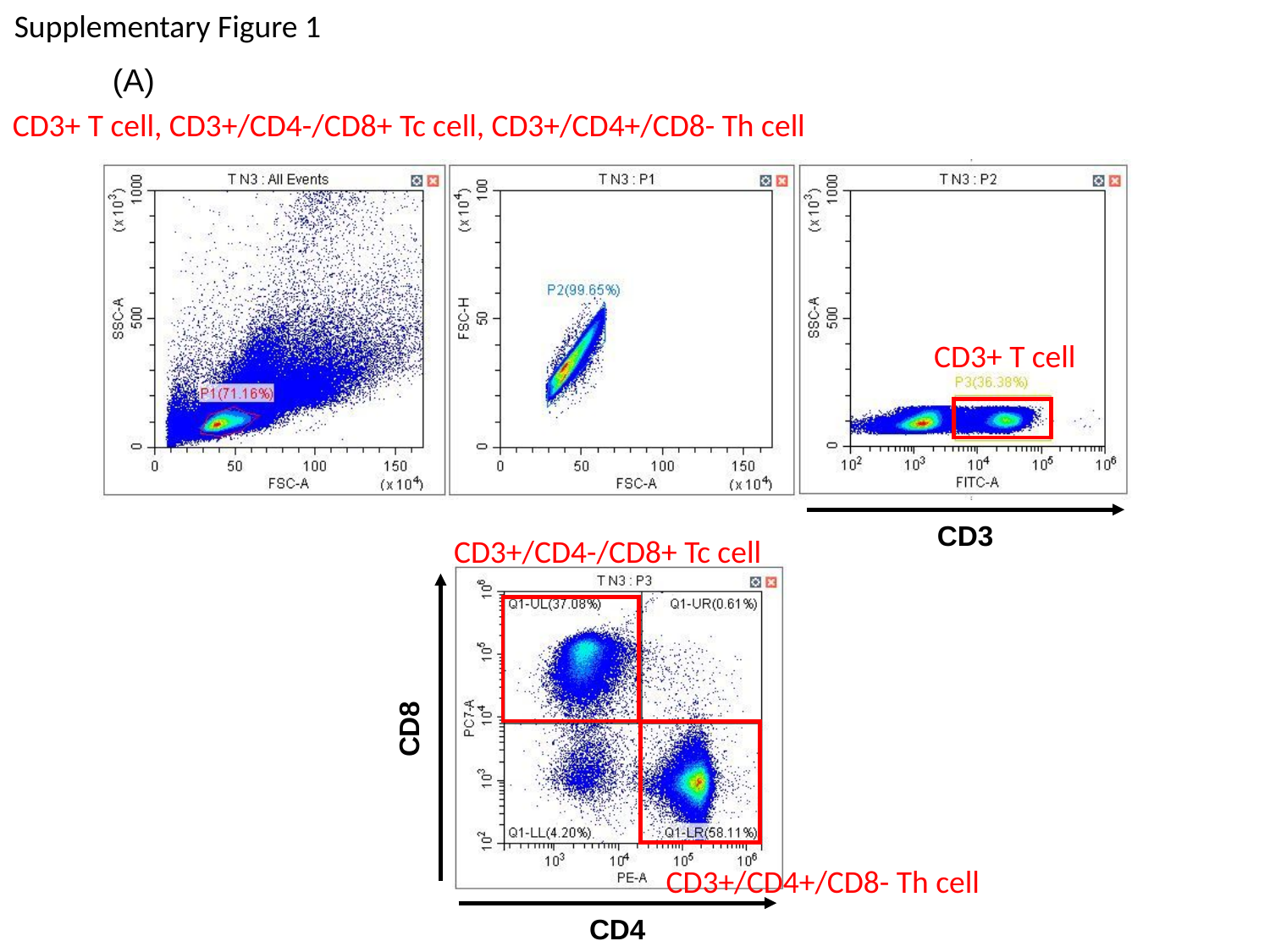

Supplementary Figure 1
(A)
CD3+ T cell, CD3+/CD4-/CD8+ Tc cell, CD3+/CD4+/CD8- Th cell
CD3+ T cell
CD3
CD3+/CD4-/CD8+ Tc cell
CD8
CD3+/CD4+/CD8- Th cell
CD4

## Slide 3
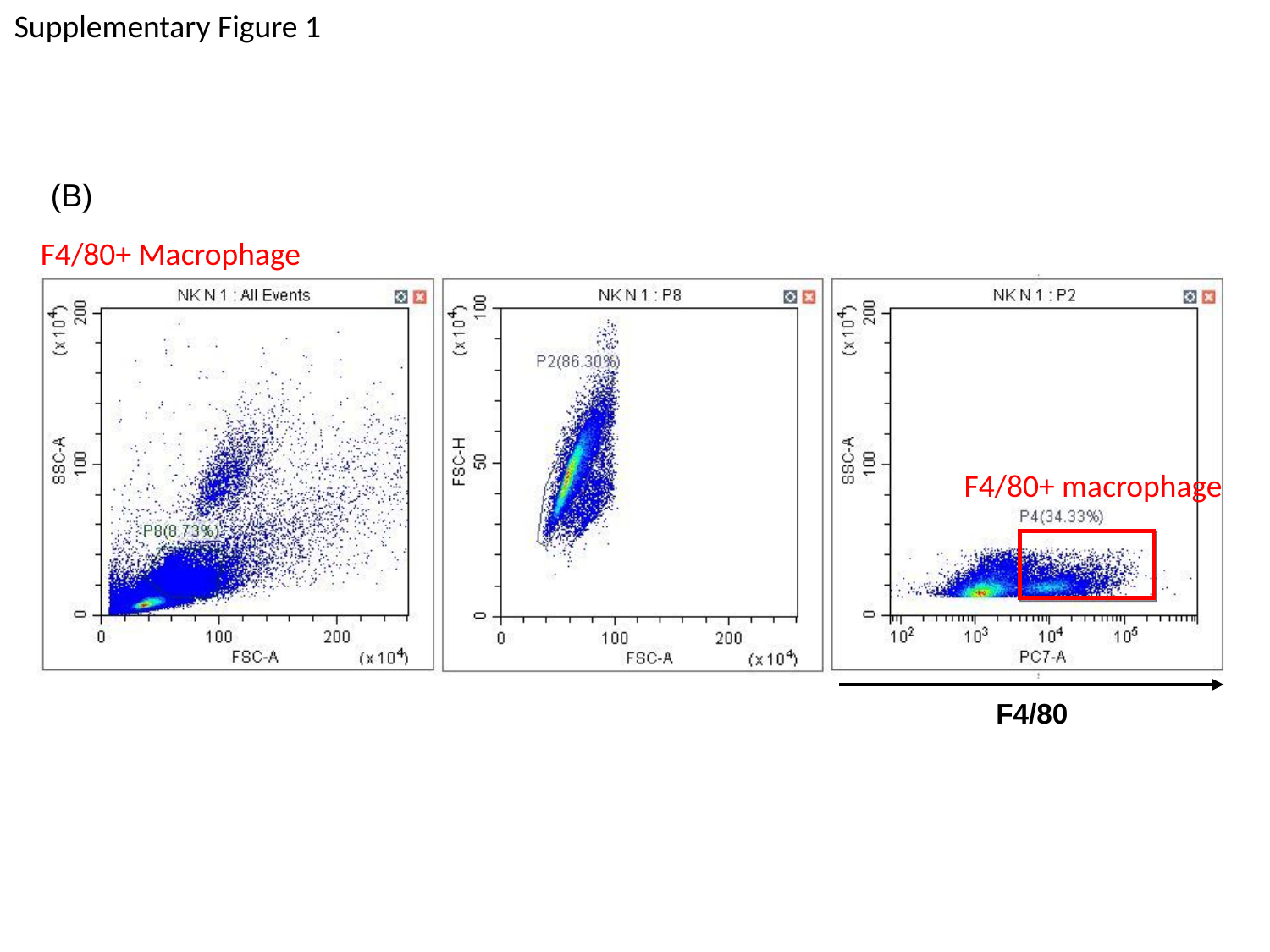

Supplementary Figure 1
(B)
F4/80+ Macrophage
F4/80+ macrophage
F4/80

## Slide 4
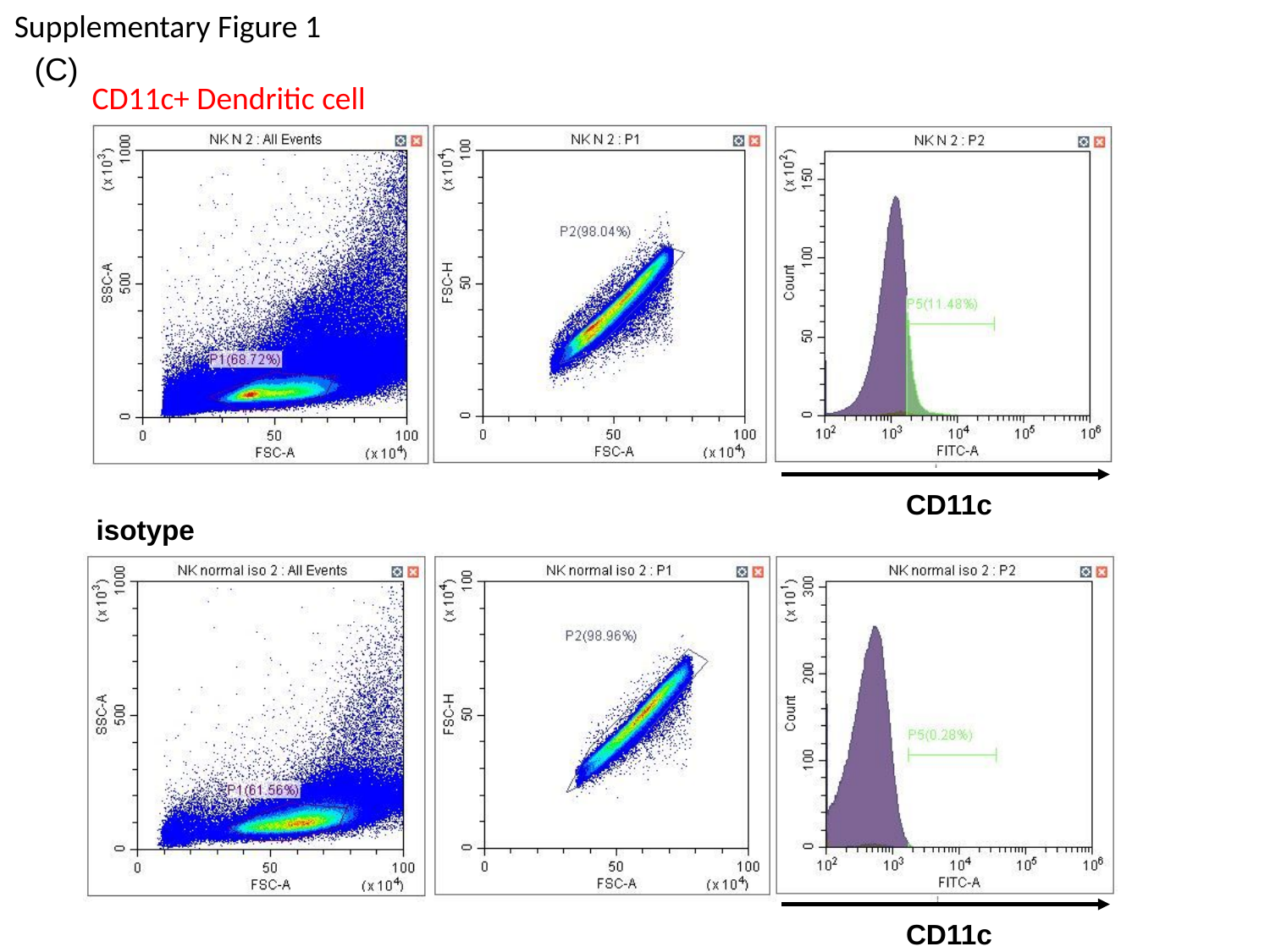

Supplementary Figure 1
(C)
CD11c+ Dendritic cell
CD11c
isotype
CD11c

## Slide 5
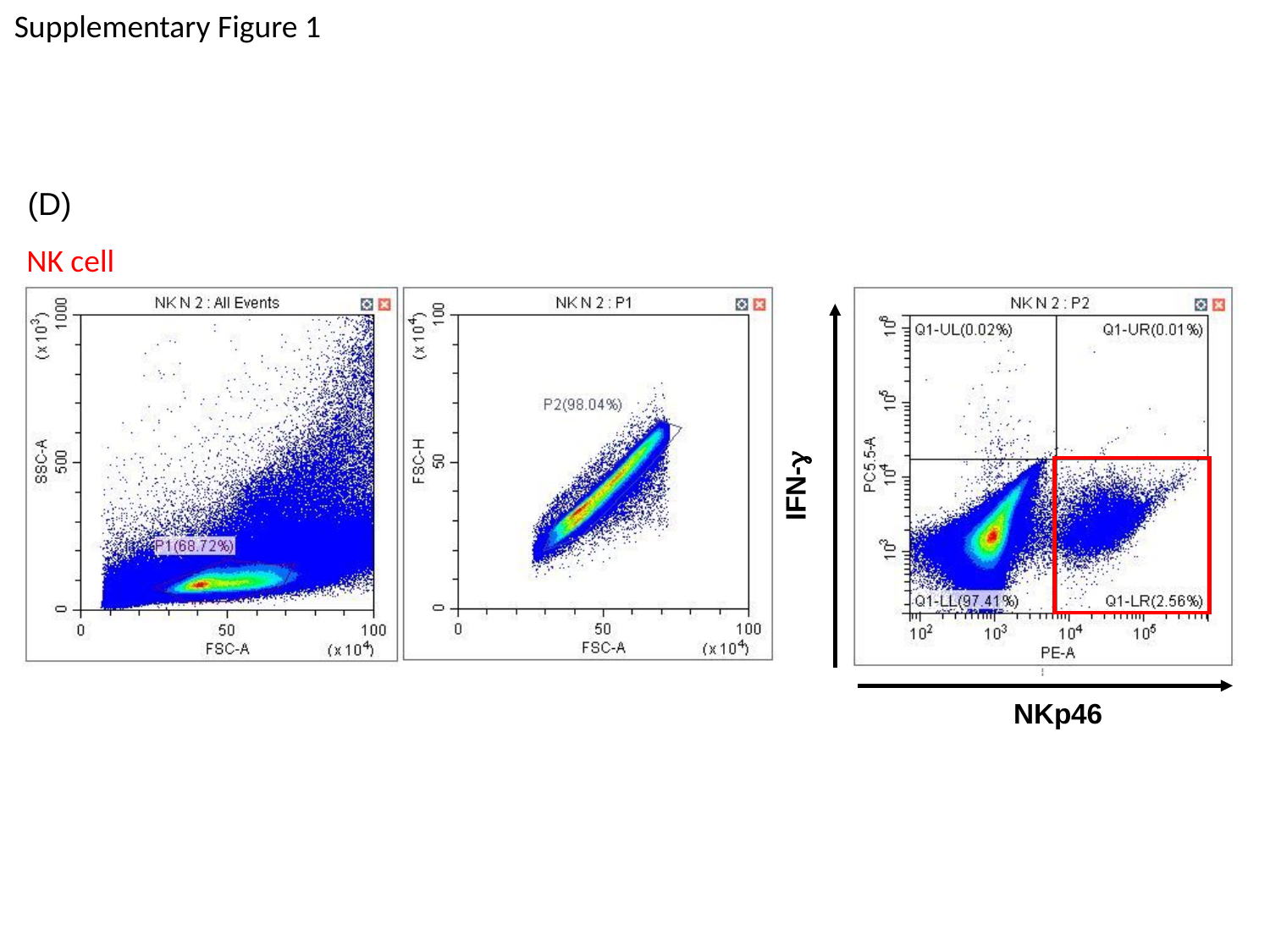

Supplementary Figure 1
(D)
NK cell
IFN-g
NKp46
